# Comparison of 8-vs-12 weeks, adapted dialectical behavioral therapy (DBT) for borderline personality disorder in routine psychiatric inpatient treatment—A naturalistic study

**DOI:** 10.1038/s41598-024-61795-9

**Published:** 2024-05-17

**Authors:** Milenko Kujovic, Daniel Benz, Mathias Riesbeck, Devin Mollamehmetoglu, Julia Becker-Sadzio, Zsofia Margittai, Christian Bahr, Eva Meisenzahl

**Affiliations:** 1 Department of Psychiatry and Psychotherapy, Medical Faculty and University Hospital Düsseldorf, Heinrich-Heine University Düsseldorf, Düsseldorf, Germany; 2 University Hospital for Psychiatry and Psychotherapy, Neurophysiology & Interventional Neuropsychiatry, Tübingen Center for Mental Health, University Hospital Tübingen, Tübingen, Germany

**Keywords:** Borderline personality disorder, Dialectical behavior therapy, Treatment duration, Depressive symptoms, BPD symptomatology, Health services, Public health, Health care, Medical research, Psychology, Human behaviour

## Abstract

Dialectical behavior therapy (DBT) is widely acknowledged as an effective treatment for individuals with borderline personality disorder (BPD). However, the optimal treatment duration within DBT remains a topic of investigation. This retrospective, naturalistic non-randomized study aimed to compare the efficacy of 8 week and 12 week DBT interventions with equivalent content, focusing on the change of BPD-specific symptomatology as the primary outcome and depressive symptoms as the secondary outcome. Overall, 175 patients who participated in DBT and received either 8 week or 12 week intervention were included in the analysis. Routine inpatient treatment was adapted from standard DBT with the modules: skill training, interpersonal skills, dealing with feelings, and mindfulness. Measurements were taken at baseline, mid-point, and endpoint. The borderline symptom list-23 (BSL-23) was used for the assessment of borderline-specific symptoms, while the Beck depression inventory-II (BDI-II) was used for the assessment of depressive symptoms. Statistical analysis was conducted using linear mixed models. Effect sizes were calculated for both measures. The results of the analysis indicated an improvement in both groups over time. Effect sizes were *d* = 1.29 for BSL-23 and *d* = 1.79 for BDI-II in the 8 week group, and *d* = 1.16 for BSL-23 and *d* = 1.58 for BDI-II in the 12 week group. However, there were no differences in the change of BPD-specific symptoms or the severity of depressive symptoms between the 8 week and 12 week treatment duration groups. Based on these findings, shorter treatment durations, like 8 weeks, could be a viable alternative, offering comparable therapeutic benefits, potential cost reduction, and improved accessibility. However, further research is needed to explore factors influencing treatment outcomes and evaluate the long-term effects of different treatment durations in DBT for BPD.

Trial registration: drks.de (DRKS00030939) registered 19/12/2022.

## Introduction

Dialectical behavioral therapy (DBT) was originally developed to treat chronically suicidal patients by Marsha Linehan^1^ and is regarded as the first choice evidence-based treatment for borderline personality disorder (BPD^2^). BPD is a severe mental illness^3^ and describes a pattern of emotional, behavioral, cognitive, and interpersonal dysregulation leading to marked distress as well as impairments in social and occupational functioning^1,4^. Furthermore, BPD is characterized by self-harming behaviour, an increased risk of suicide^5^ and high rates of axis-I comorbidities, with mood disorders being the most prevalent^6^. In addition, high comorbidities are shown for eating disorders, substance abuse, post-traumatic stress disorder, and personality disorders^7^. While recent long-term studies show that remission of symptoms was sustained over time in almost one half of the affected individuals, social integration was significantly worse^7^. According to population-based studies, the prevalence of BPD ranges from 0.7 to 4.5%^8^, with a lifetime prevalence of 5.9%^9^. This suggests that in Germany, there are an estimated 500,000 to 1,000,000 individuals affected by this condition^10–12^. Besides, BPD patients show a high prevalence among psychiatric (in-)patients^4^.

Furthermore, the outpatient and inpatient care situation for BPD in Germany can be considered insufficient^10^. Thus, an estimated 700 inpatient places are available^11^ and therapists in inpatient and outpatient care are often not specifically trained or may even refuse treatment in some cases^10^. Furthermore, Iliakis et al.^13^ estimated a ratio of 1:1102 of specialized, certified therapists in relation to the annual number of BPD patients to be treated. Beyond the individual burden caused by BPD, the economic burden by means of high mental health care costs—mainly driven by repetitive hospital stays^3,14,15^—is immense. Direct and indirect costs are estimated to result in numbers of up to € 40.000 per case and year depending on factors included and approach^3^. Accordingly, there is a need for specialized hospitals to conduct the complex management of chronic diseases as well as to provide the necessary resources.

According to several treatment guidelines for BPD, psychotherapy is considered first-line therapy^16,17^. Considering the German guideline for BPD^16^, DBT shows the best evidence and is recommended particularly when the treatment’s primary outcome is the reduction of severe self-harming behaviors (including suicidal behaviors). Furthermore, manualized disorder-specific psychotherapy programs such as DBT, mentalization-based psychotherapy (MBT)^18^ and schema therapy^19^ have been found to be effective^20^. Also, Bohus^7^ stated that disorder-specific treatment in the case of BPD in comparison to unspecific treatment seems to lead to further improvements, e.g. lower suicide rates. DBT is a modularized, individual and group-based skills training consisting of four key elements: mindfulness, distress tolerance, emotion regulation and interpersonal competence^1^. Additionally, DBT has since been advanced to include other important components, such as self-esteem, as BPD is often associated with dysfunctional self-concepts^21^. The efficacy of DBT for BPD has been proven in several randomized controlled trials with different designs^22,23^. These findings are supported by recent systematic reviews and meta-analysis showing that DBT is effective in reducing BPD specific symptoms and superior compared to treatment-as-usual (e.g.,^23,24^). According to Snoek et al.^25^, DBT offers a more favorable cost-effectiveness as compared to cognitive behavioral therapy (CBT) or other treatments, such as weekly individual therapy or psychoeducational groups.

While many studies have investigated the efficacy of psychotherapeutic treatments, only a few have focused on the general framework or organizational conditions of psychotherapeutic treatment (such as the duration of treatment, or inpatient vs. outpatient setting) and their effectiveness^26^. Knowledge about the influence of these contextual factors on therapy outcomes is still somewhat limited. Originally, DBT was developed as an outpatient treatment as hospitalization might decrease patients’ ability to learn effective coping strategies for their daily lives^1^. According to Van Swearingen and Lothes^27^, the standard version of DBT within outpatient settings should require approximately 1 year. However, BPD patients often require more intensive care and show a high prevalence in inpatient settings^4^. In addition, Bloom et al.^28^ argue that the outpatient setting cannot adequately be provided for all BPD patients as well as that outpatient treatment staff might be feeling overwhelmed when dealing with BPD. Consequently, DBT was adapted for inpatient settings^29^.

One of the first studies to examine DBT in the inpatient setting was performed by Bohus et al.^30^, who adapted this type of DBT program by Swenson et al.^31^ for the inpatient setting in Europe. The treatment lasts about 3 months^30^. Yet, inpatient treatment programs for BPD show variation in the duration and content^28^. Compared to the 1 year DBT originally scheduled by Linehan^1^, abbreviated inpatient and outpatient implementations of DBT were studied with different variations of duration, ranging from very short 5 day intensive group-based DBT-skills training to longer-term 6 month programs^26,32^. Despite heterogeneity in the duration of inpatient treatment programs a survey by Richter et al.^11^ found that among 42 German hospitals and day clinics about half of the clinics set the treatment duration a priori to 12 weeks. Furthermore, Bloom et al.^28^ found the modal duration of inpatient DBT to be 3 months. Consequently, it can be assumed that 3 months (12 weeks) is the most common duration of inpatient DBT in Germany and can be seen as the standard duration in the inpatient setting.

Based on studies examining the effects of duration of DBT treatments in the inpatient setting, research has also suggested that even short versions of DBT could be (equally) effective in reducing BPD-specific symptoms. A study conducted in a German hospital found small to medium effect sizes regarding the reduction of BPD symptoms within a treatment duration between 8 and 12 weeks^33^. This is also one of the few naturalistic studies that has been conducted within routine care^26^. Other studies have shown different beneficial effects for shorter treatment durations. For instance, 25% of patients seemed to refrain from self-harm within the first week of therapy^34^. Additionally, Probst et al.^35^ showed that a 5 weeks inpatient DBT therapy showed a significant reduction in BPD specific symptoms and improved emotion regulation. In addition, results indicate that symptom and functional improvement for shortened therapies were stable at 5 year follow-ups with annual measurements and readmission rates remained low after treatment completion^26,27^. Regarding other setting conditions, recent research suggests that DBT-inpatients may benefit more than outpatients regarding self-esteem, distress, and quality of life^26^.

Although the evidence base is somewhat sparse and further research is urgently needed, it suggests that shorter (inpatient) DBT may be as effective as a longer treatment. Moreover, shorter treatment duration is associated with potentially different advantages like reduced dropout rates^26^ or reduced health care and societal costs. A recent meta-analysis found that dropout rates in psychotherapies for BPD are generally high, ranging between 20 to 30%^36^. According to Iliakis et al.^36^ main reasons for dropping out were treatment dissatisfaction, exclusion from treatment, insufficient motivation, as well as life events or changes in life situation. Likewise, Iliakis et al.^36^ suggest that abbreviated treatment programs could have an impact on patient motivation and satisfaction, thereby increasing adherence and commitment to therapy as well as reducing dropout rates. Furthermore, a shortened treatment duration enables an earlier treatment completion and should lead to a greater benefit for BPD-patients in general as more patients could participate in disorder-specific treatments within the same period and facility^26^. Also beneficial is an earlier treatment response, e. g. faster reduction of self-harming behavior and earlier return to ‘real life’ leading to (earlier) occupational as well as leisurely activities.

### Aims of the study

Consequently, we argue that an adequate adjustment of ‘standard’ DBT in terms of shortened treatment duration of 8 weeks compared to 12 weeks within inpatient setting might deliver significant benefits for patients with BPD while maintaining the efficacy of (longer) DBT-programs. Accordingly, we hypothesize that a shortened 8 week DBT is comparable regarding efficacy to the standard 12 week program (both with equal content) in routine clinical psychiatric inpatient treatment. The primary outcome of the study was the change in BPD-specific symptoms, while the secondary outcome was the change in depressive symptoms.

## Methods

### Procedures

The study was conducted between August 2019 and September 2021 at a specialized ward for patients with BPD at the LVR-clinics Dusseldorf, department of psychiatry and psychotherapy at the Heinrich–Heine-university, Dusseldorf. Each potential patient receives a pre-admission interview prior to acceptance. After admission within routine clinical treatment, patients with BPD were offered a DBT program as obligatory to continue inpatient treatment. Generally, the treatment plan was scheduled for 8 weeks. From August 2020 to March 2021 an adaptation was implemented to offer an extension of four more weeks (i.e. 12 weeks in total) to patients showing high commitment and motivation to deepen and improve knowledge and coping skills. The decision to extend was made during the sixth week by the clinical assessment of the treatment team. This applied to patients who were engaged and participated effectively in the program. Accordingly, patients who extended the treatment up to 12 weeks had the opportunity to repeat and practice. Nevertheless, beyond time and treatment session extension, there were no differences regarding contents or skills training provided. The difference in treatment duration resulted in two separate samples of patients who received the same DBT treatment, but were treated for either 8 or 12 weeks. For all patients, the routine clinical treatment also comprises occupational therapy, sports/physical activity therapy, music therapy, and psychiatric care including psychotropic drug treatment, which should be administered as low as possible. All data were collected within the routine treatment and analyzed post hoc. Assessments included in the analyses considered baseline (prior treatment), midpoint (after 4 or 6 weeks respectively) and after treatment (week 8 or 12 respectively). Due to the routine care setting, both treatment conditions were provided by the same personnel. Initial assessments including diagnosis were blind to treatment condition, as group allocation took place in the sixth week of DBT-treatment at the earliest. Assessments after week 8 were not blind to treatment condition, however assessors i.e. patients (due to self-assessment instruments) as well as treating personnel were unaware of the hypothesis. Adherence (of therapists) to (DBT-) manual was not assessed. All participants have given informed consent to anonymized analyses as the standard procedure associated with inpatient treatment. Before retrospective data collection, an ethics vote was requested from the Medical Faculty Ethics Committee of Heinrich Heine University, Dusseldorf, which was approved on 1 February 2022 (reference number: 2021-1693). All methods were performed in accordance with the relevant guidelines and regulations.

### Inclusion/exclusion criteria and sample

All patients with a diagnosis of BPD according to DSM-5 criteria^37^, which was assured by SCID-II (meeting at least five criteria on the BPD scale^38^,) and additionally confirmed by means of a clinical diagnosis according to ICD-10^39^ were included in the trial and analyses. Also, diagnosis of depression and further mental disorders were confirmed using Diagnostisches Kurzinterview bei psychischen Störungen (mini-DIPS OA^40^,). Trained clinicians that were either psychotherapists or psychotherapists in training conducted the diagnostic process. Training in SCID-II assessments are part of the routine clinical management at our facility, however no formal reliability checks were conducted. Furthermore, patients had to be at least 18 years old, commencing DBT treatment and having at least baseline assessment in the borderline symptom checklist 23 (BSL-23^41^;). In the study, all comorbidities were allowed except for disorders within the schizophrenia spectrum and addiction disorders, which were considered exclusion criteria. Overall, 175 patients participated, 153 patients under an 8 weeks treatment condition and 22 patients under 12 week condition.

### Primary outcome: BSL-23

The BSL is a self-rating instrument for assessing typical symptoms associated with the BPD^41^. The items address both diagnostic criteria, such as affective instability and self-harming behavior, as well as borderline-typical empirical findings regarding self-criticism, trust issues, emotional vulnerability, and feelings of shame, loneliness, and helplessness^42^. The BSL is available in long and short versions. The long version consists of 95 items while the short version assesses the symptoms with only 23 items. The BSL-23 is used for measuring the borderline specific symptoms 1 week prior to the assessment^41^. Participants’ ratings are given on a Likert scale from 0 (not at all) to 4 (very strong). BSL-23 has proven to have sufficient psychometric properties regarding validity and reliability^41^. In the present study, the standardized percentile rank of the test was analyzed.

### Secondary outcome: BDI-II

The revised Beck depression inventory (BDI-II) is a self-report questionnaire designed to measure the severity of depression in individuals^43^. The BDI-II consists of 21 items where individuals can rate the severity of their symptoms on a scale from 0 to 3, whereas higher scores indicate more severe depression. The BDI-II has high internal consistency as well as good validity^44^.

### DBT treatment

The offered modularized DBT program according to Linehan’s manual adapted for inpatient treatment in Germany by Bohus and Wolf–Arehult^45^, contains the modules: skill training, interpersonal skills, dealing with feelings, and mindfulness. The self-esteem module is not provided. The typical treatment comprises the following obligatory components: each patient receives individual psychotherapeutic sessions (1–2 per week), DBT-based skills training (group: 2 per week), mindfulness-based group therapy (1 per week), psychoeducation about DBT and BPD (1 per week), and “tools” group (consolidation of elements taught in DBT such as emotion analysis, 1 per week). The program was designed for 8 weeks, accordingly all patients were provided with the opportunity to complete the four modules offered. Patients in the 12 week group were not provided with more content in the program, but were able to use the additional time to repeat the content. To ensure adherence to the DBT manual, all staff members in direct patient contact from various professional backgrounds, including medical personnel, psychotherapists, nursing staff, occupational therapists, and others, underwent training in all modules (basis- and skills-modules) provided by the Dachverband DBT e.V. Certified personnel from the DBT association conducted these training sessions consisting of six modules of a total of 96 h of instruction.

### Statistical analysis

Routine data was analyzed post hoc. Group differences (8 vs. 12 weeks) in primary (BSL-23) and secondary (BDI-II) endpoints were analyzed by linear mixed models repeated measurement with group, time (baseline, mid- and endpoint) and group*time-interaction as fixed effects and patient as random effect to deal adequately with missing values (intention-to-treat analyses). In addition, baseline scores (BSL-23 or BDI-II respectively) were included as covariates. To control for potential ‘historical’ effects (prior March 2021, 8 week and 12 week DBT was offered, after March 2021 only 8 weeks DBT; thus, after March 2021 n = 52 patients, i.e. 34% of all 153 patients with 8 weeks-DBT, were treated for 8 weeks whereas no patient for 12 weeks) all analyses were also conducted regarding a three-group comparison (8 weeks pre March 21 vs. 8 weeks post March 21 vs. 12 weeks). Effect sizes were calculated using the estimated means of the linear mixed model including baseline as covariate divided by the pooled standard deviation of both groups at baseline. To test for pre-treatment differences, Chi-square-tests were conducted for categorical measures and *t*-tests (two-group comparisons) or ANOVAs (three-group comparisons) for metric measures. In addition, the potential confounding effect of group differences in routine treatment with psychotropic drugs was analyzed regarding the kind of drugs, amount of drugs in ‘days applied’ and percentage of hospital days with drugs (the patients of the 12 week group had naturally a significantly longer hospital stay, thus the percentage was calculated). Due to the computer based documentation system of the hospital, drug dose could not be assessed. Given the naturalistic and retrospective design of the study and especially the evolving divergent group sample sizes (n = 153 for 8 week vs. n = 22 for 12 week DBT) statistical requirements to examine a more appropriate non-inferiority hypothesis (especially balanced groups for sound parameter estimates) were unfortunately not given. All analyses were conducted using IBM SPSS statistics version 29.

## Results

### Descriptives

The mean age of the participants in the 8 week program was 28.3 (*SD* = 8.6), whereas participants in the 12 weeks program were on average 24.7 (*SD* = 7.1) years old. This difference was significant, *t*(173) = 1.9, *p* = 0.036. Regarding sex proportions both samples showed no significant differences, Χ^2^(1) = 0.54, *p* = 0.58. The proportion of females in the 8 week group was 79.7% and 86.4% in the 12 weeks condition. While the cumulative days of inpatient treatment in the 8 weeks group averaged 55.6 days (*SD* = 12.5), individuals in the 12 week group spent an average of 75.5 days (*SD* = 12.2) in the hospital. Accordingly, the 12 week group showed a discrepancy in terms of the intended average duration. Table 1 shows the observed means of BSL-23 and BDI-II as well as their standard deviations for both groups regarding all three measurement points. At baseline, BSL-23 and BDI-II were not significantly different between treatment groups (*p* = 0.33 and 0.12 respectively). In addition, comorbidity with (other) mental disorders according to ICD-10 diagnosis was not significantly different between groups (see Table S1 in supplement).

**Table 1:** Observed mean percentile rank and standard deviations of BSL-23 and BDI-II for both groups, 8 weeks and 12 weeks.

	BSL-23^a^		BDI-II^b^	
	8 weeks	12 weeks	8 weeks	12 weeks
Baseline^c^	56.8 (25.5)	62.5 (25.7)	36.8 (9.8)	40.3 (10.2)
Mid	41.1 (27.7)	37.9 (25.0)	28.4 (12.4)	28.9 (12.8)
End	25.3 (26.2)	30.1 (26.2)	19.2 (12.6)	23.4 (14.7)

At baseline sample size in the 8 weeks group was n = 153 and n = 22 in the 12 weeks condition for both tests.

^a^Mid: sample size was n = 122 for 8 weeks and n = 18 for 12 weeks. End: sample size of n = 114 (8 weeks) and n = 22 (12 weeks).

bMid: sample size was n = 126 for 8 weeks and n = 18 for 12 weeks. End: sample size of n = 113 (8 weeks) and n = 22 (12 weeks).

^c^Group comparisons at baseline: *p* = 0.33 for BSL-23 and *p* = 0.12 for BDI-II.

### Mixed linear models analysis

#### BSL-23: primary outcome

**Figure 1:**
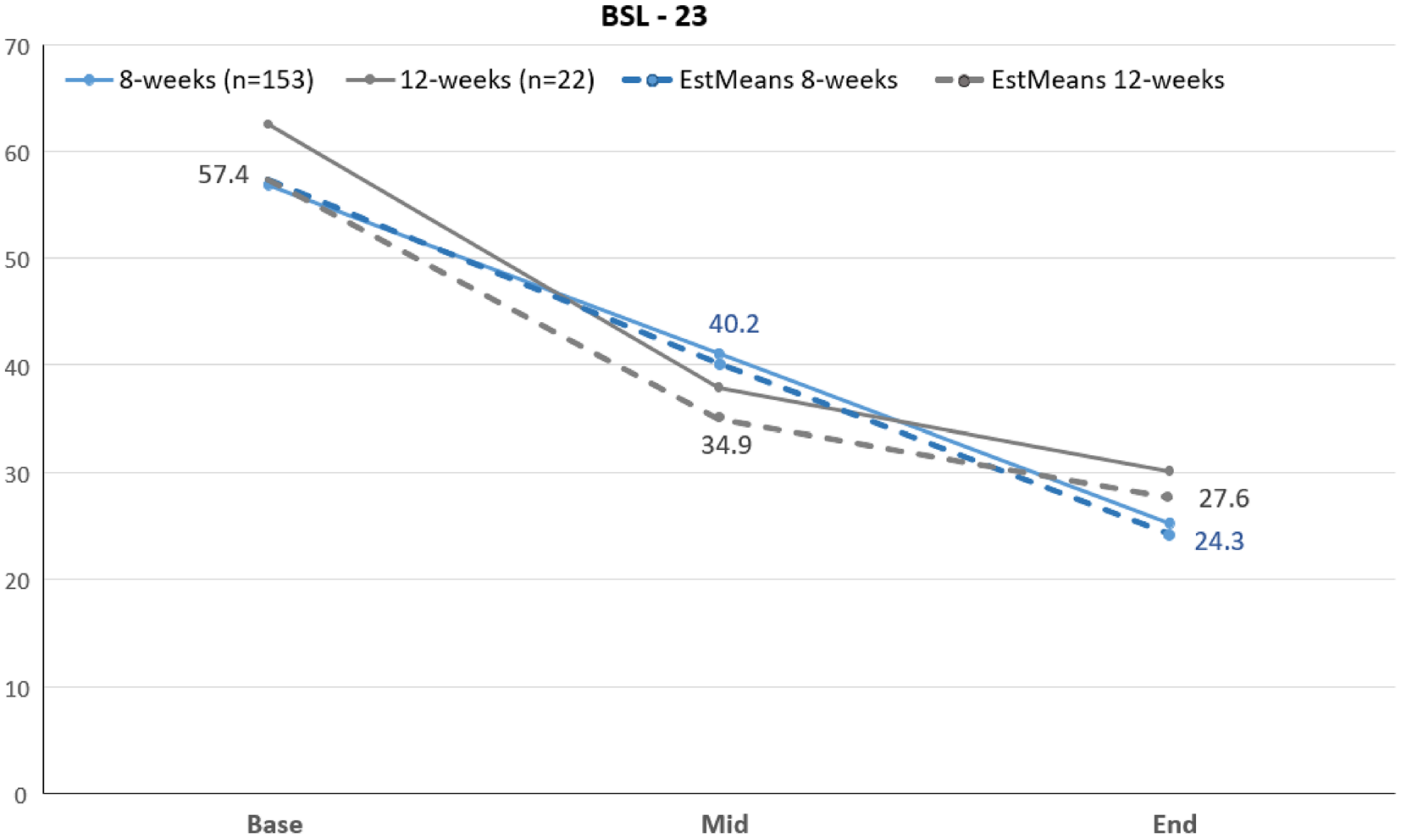
Observed and estimated percentile ranks regarding the BSL-23 for both groups on all assessment time points: Base, mid (after 4 weeks for 8 weeks treatment and after 6 weeks for 12 weeks treatment) and end (after 8 weeks for 8 weeks treatment and after 12 weeks for 12 weeks treatment).

**Figure 2:**
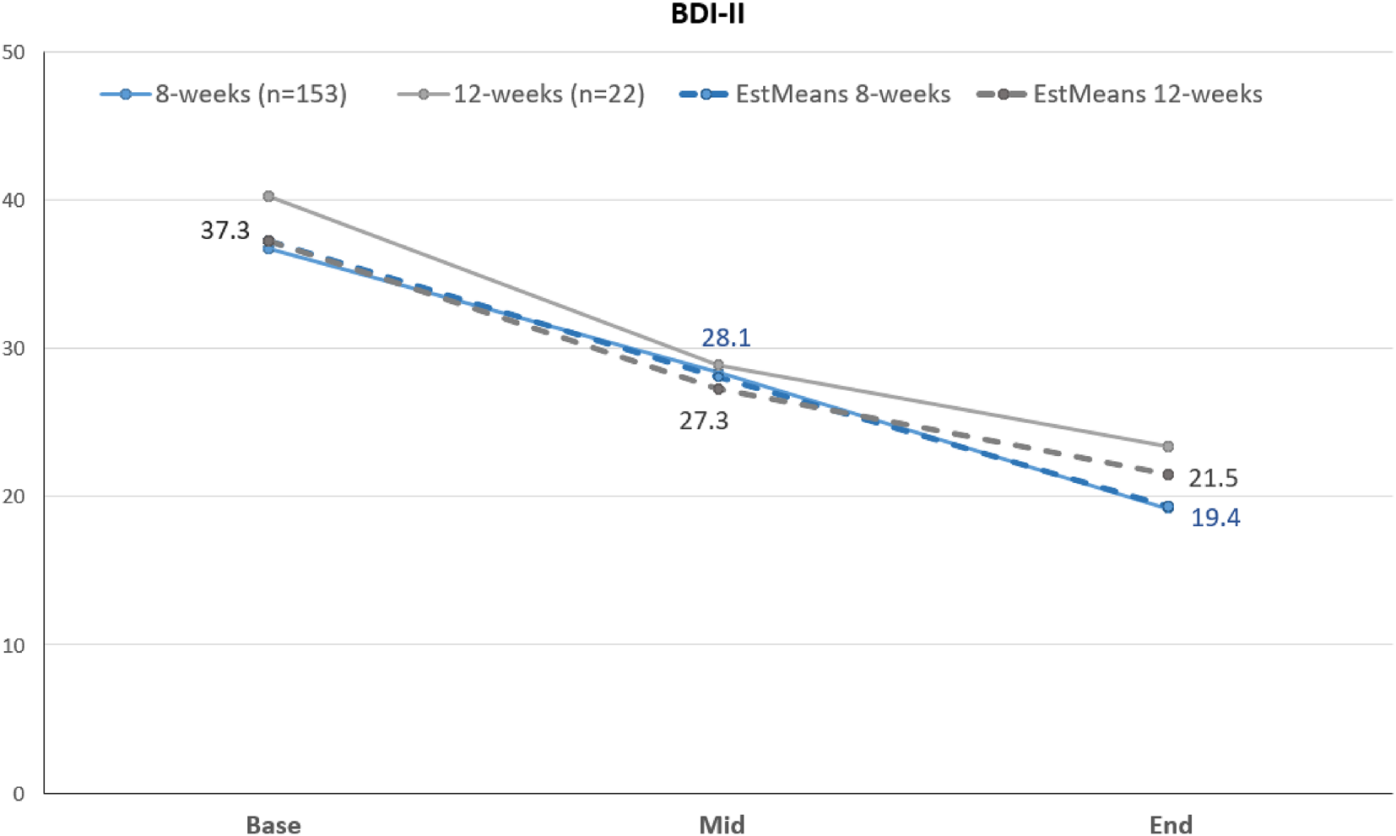
Observed and estimated sum scores for BDI-II for both groups over time; ‘Mid’ = after 4 weeks for 8 weeks treatment and after 6 weeks for 12 weeks treatment; ‘End’ = after 8 weeks for 8 weeks treatment and after 12 weeks for 12 weeks treatment.

In this study, we used mixed linear models to compare two groups (8 weeks vs. 12 weeks DBT) with respect to changes in BSL-23 as the primary outcome. Table 2 shows the estimated mean percentile ranks for BSL-23 comparing 8 weeks against the 12 weeks treatment. Figure 1 depicts the observed and estimated mean percentile ranks for all three measurement time points comparing 8 weeks with 12 weeks of DBT. As Fig. 2 shows there is a decline in borderline specific symptoms over time, this main effect was significant, *F*(1, 120.11) = 19.45, *p* < 0.001. Therefore, borderline specific symptoms reduced significantly over time in both groups. The main effect of the group was not significant, *F*(1, 161.43) = 0.04, *p* = 0.85, as well as the interaction of group and time, *F*(1, 120.09) = 2.66, *p* = 0.11. Accordingly, groups showed no differences regarding BSL-23 at any time nor in reduction over time. With respect to single time comparisons, only the comparison of mid to end was not significant for twelve weeks, *p* = 0.29. Likewise, the three-group-comparison (8 weeks pre March 21 vs. 8 weeks post March 21 vs. 12 weeks) yielded comparable results.

**Table 2:** Estimated mean percentile ranks and 95% confidence intervals of BSL-23 for both groups, 8 weeks and 12 weeks, using mixed-effects models with baseline as covariate.

	BSL-23			
	8 weeks	95% CI	12 weeks	95% CI
Baseline^a^	57.4	–	57.4	–
Mid^b^	40.2	[36.4;44.0]	34.9	[24.9;45.0]
End^c^	24.3	[19.8;28.7]	27.6	[17.0;38.3]

^a^Due to including baseline BSL-23 score as covariate no 95% CI evolved.

^b^After 4 weeks for 8 weeks treatment and after 6 weeks for 12 weeks treatment.

^c^After 8 weeks for 8 weeks treatment and after 12 weeks for 12 weeks treatment.

### BDI-II: secondary outcome

Also for the secondary outcome regarding the BDI-II a linear mixed model was conducted. Table 3 shows the estimated means and 95% confidence intervals for BDI-II for 8 weeks and 12 weeks treatment groups. As can be seen in Fig. 2 BDI-II scores significantly improved over time, *F*(1, 121.06) = 31.42, *p* < 0.001. Likewise, as for BSL-23, there was no significant main effect of the group on BDI-II, *F*(1, 161.39) = 0.08, *p* = 0.78, and no significant interaction of group and time, *F*(1, 121.02) = 1.30, *p* = 0.26. Regarding single comparisons, all specific time effects were highly significant (*p* < 0.001) except for mid to end for the 12 weeks group, *p* = 0.040. Regarding the three-group-comparison (8 weeks pre March 21 vs. 8 weeks post March 21 vs. 12 weeks), besides a significant time-effect (*p* < 0.001) and a non-significant group*time-interaction (*p* = 0.32), a significant group-effect (*p* = 0.006) evolved. However, as post-hoc comparisons indicate, the groups of ‘8 weeks pre March 21’ shows a significantly higher BDI-II reduction as compared to the ‘8 weeks post March 21’ at mid- as well as the endpoint, but both groups were not significantly different from the ‘12 weeks’ group (see Fig. S1 in the supplement).

**Table 3:** Estimated means and 95% confidence intervals of BDI-II for both groups, 8 weeks and 12 weeks, using mixed-effects models with baseline as covariate.

	BDI-II			
	8 weeks	95% CI	12 weeks	95% CI
Baseline^a^	37.3	-	37.3	-
Mid^b^	28.1	[26.3;29.9]	27.3	[22.5;32.0]
End^c^	19.4	[17.3;21.5]	21.5	[16.5;26.6]

^a^Due to including baseline BDI-II score as covariate no 95% CI evolved.

^b^After 4 weeks for 8 weeks treatment and after 6 weeks for 12 weeks treatment.

^c^After 8 weeks for 8 weeks treatment and after 12 weeks for 12-weeks treatment.

### Effect sizes

**Figure 3:**
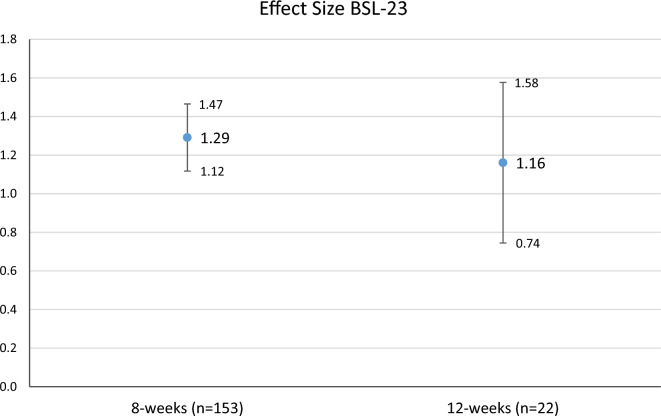
Effect sizes and 95% CIs from the beginning of the treatment to the end of the treatment separately for 8- and 12 weeks groups for BSL-23.

**Figure 4:**
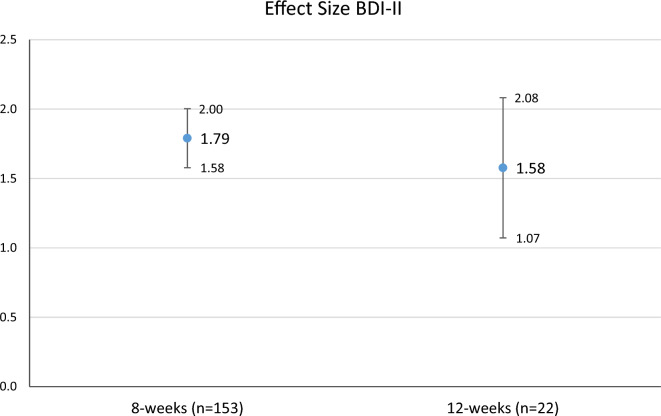
Effect sizes and 95% CIs from the beginning of the treatment to the end of the treatment separately for 8- and 12 weeks groups for BDI-II.

Effect sizes for overall symptom reduction were calculated for both BSL-23 and BDI-II values. Figure 3 shows the effect sizes for the BSL-23 and Fig. 4 for BDI-II. As can be seen the overall effect size of treatment on BSL-23 and BDI-II were high (reduction greater than one standard deviation of baseline scores) for the 8 week and 12 week group. Nevertheless, 95% CIs indicate that there were no significant differences between 8 and 12 weeks. Regarding the three-group-comparison (8 weeks pre March 21 vs. 8 weeks post March 21 vs. 12 weeks), 95% CIs likewise indicate a (significant) higher effect for the group ‘8 weeks pre March 21’ compared to the ‘8 weeks post March 21’ in BDI-II reduction, but again, both groups were not significantly different from the ’12 weeks’ group (see Fig. S2 in the supplement).

### Psychotropic drugs

Analyses regarding group differences in treatment with psychotropic drugs within the treatment phase (see Table S2 in supplement) revealed only slight, however significant differences in treatment with mood stabilizers and sleeping drugs. Whereas the proportions of patients with such drugs was not significantly different, the mean percentage of days with such drugs (related to total treatment duration) differs (mood stabilizer: *M*/*SD* = 4.1%/8.6 for 8 weeks group and 0.1%/0.4 for 12 weeks group, *p* < 0.001; sleeping drugs: 3.3%/12.2 for 8 weeks group and 0.1%/0.3 for 12 weeks, *p* < 0.001). Regarding treatment with antidepressants, antipsychotics, benzodiazepines or other psychotropic drugs (predominantly stimulants for ADHD treatment) no significant differences evolved.

## Discussion

The current retrospective, naturalistic study examined the efficacy of an 8 weeks DBT inpatient treatment in comparison to a 12 weeks DBT treatment within routine care. We found no differences regarding the reduction in borderline specific symptoms as well as the severity of depressive symptoms between both groups. Furthermore, both treatment groups showed high effect sizes regarding BPD-specific symptoms as well as depressive symptoms. Therefore, we conclude that an 8 week treatment was as effective as a longer 12 weeks treatment.

The fact that we found no difference between the two groups is even more surprising given a possible selection bias for the 12 weeks group as the patients were marked as highly motivated and were therefore given the opportunity to deepen their DBT knowledge and skills for another 4 weeks. One possible explication was given by Seow et al.^26^, who found no difference between a (very) short intensive DBT skills program of 5 days and a 12 weeks DBT treatment. Based on the good-enough level model^46,47^, it is argued that treatment is a mutual process between patients and practitioners, whereby therapy completes in case of sufficient improvement^26^. A lower dose regarding overall treatment length could lead to a higher effort, commitment and adherence to achieve a symptom reduction in a shorter period of time^26^. Thus, this would be a possible explanation, as in the current study the decision to extend to 12 weeks was made already in the sixth week of the treatment.

The overall reduction of borderline specific and depressive symptoms in inpatient settings using DBT is in line with Bloom et al.^28^, who suggested that inpatient DBT could facilitate the treatment of BPD. Moreover, a shorter DBT implementation than the most commonly used 12 week duration in the inpatient setting was equally effective in reducing borderline-specific symptoms. In comparison to the literature, the estimated effect sizes in the current studies were larger. Probst et al.^35^ found effect sizes between *d* = 0.38 and *d* = 0.47 for intention to treat analysis and completers using the BSL-23 while conducting a 5 week inpatient DBT treatment. Also, Probst et al.^35^ compared their effect sizes with effects reported in previous studies. Those effect sizes varied between* d* = 0.13 and *d* = 1.40^35^. In addition, Seow et al.^26^ also had lower BSL-23 values in the inpatient setting, as well as Herzog et al.^33^ found smaller effect sizes for BSL-95. One possible reason could be that severity of borderline specific symptoms was higher as the observed mean percentage ranks were *M* = 56.8 for 8 weeks and *M* = 62.5 for 12 weeks treatment, compared to a mean percentage rank of *M* = 43 which corresponds to the mean raw score of 1.9 found by Probst et al.^35^. Furthermore, we had a longer treatment duration than Probst et al.^35^; 5 weeks. Due to our results, a significant symptom reduction also evolved between week 4 and week 8. Nevertheless, as stated above, a longer duration must not always contribute to efficacy as we did not find higher effect sizes for the 12 weeks treatment and found higher effect sizes compared to Bohus et al.^48^ although we had a shorter treatment duration.

Although we found that even shorter 8 weeks of treatment showed a significant reduction in BPD symptoms, several studies have shown that there is variation in the duration and even content of inpatient DBT treatments^3,26,28^. While 12 weeks was the most common duration of therapy, our results might indicate, that it does not seem necessary to use this duration. Especially since patients are out of their daily routine for a quarter of a year, making it more difficult to pursue their goals in real life and thus prevent social and functional decline. This would be consistent with the argument to establish DBT therapy primarily in the outpatient setting^1^, although this is not always feasible^28^. For this reason, there is a need to focus on what duration of therapy is appropriate and to strike a balance between costs and benefits. On the one hand, both health care and individual including the social costs of the therapy have to be considered, on the other hand, the short and long-term efficacy of the therapy has to be in focus. In this regard, treatment adherence and dropout rates are essential factors contributing to efficacy. Several different results show that shorter treatment duration contributes to better adherence and lower discontinuation rates^36,49^. As psychiatric therapy shifts to an individually adapted treatment (e.g. ^33,50^,), the duration of treatment might be considered also a variable parameter regarding personalization. Given the fundamental changes for diagnosis of personality disorders in ICD-11 which will the future (obligatory) diagnosis system in German health care, the severity categorization might also be a relevant parameter^51^. In addition, predictive models might be used to make personalized recommendations regarding the optimal therapy^33^. Therefore, the optimal treatment duration length should be considered when designing further treatment programs. Due to our results, 8 weeks of treatment seems (highly) effective, however, should be further evaluated in a larger prospective and randomized study with a longer observational period (e.g. 1–2 years) and long-term data.

### Limitations

However, consistent with Bloom et al.^28^, results remain difficult to compare as studies to date have contrasted different implementations of DBT in the inpatient setting. The implementation (especially duration and content) has not been compared in a standardized way so far. In the future, a more standardized comparison would be recommended.

One major limitation is that our study was not a randomized controlled trial comparing 8 weeks versus 12 weeks. Also, the current study lacks a control group like treatment-as-usual. Therefore, internal validity is limited. In addition, the 12 week treatment group sample was small (*n* = 22), therefore, conclusions are limited. Likewise, as the group allocation was not randomized a selection bias must be assumed regarding the 12 week period, as it was administered to particularly motivated patients. With respect to the observational design and routine data collected, another limitation arises; as described, there might be unaware influencing factors with respect to the therapy program during the period examined. Accordingly, an additional comparison was conducted controlling for time dependent influences, and the findings are presented in the supplement. Astonishingly, we found some differences in the reduction of depressive symptoms in the three-group comparison, however only between the 8 week treatment prior vs. post March 2021. Since no differences in DBT or drug treatment as well as setting structure took place, we assume that this might be an effect of the COVID-19 pandemic in Germany leading to a (further) increase especially in depressive symptoms and psychiatric patients^52^.

Also, we found minor differences regarding treatment with psychotropic drugs between the 8 week and 12 week conditions. Treatment with sleeping drugs and mood stabilizers was slightly more often in the 8 weeks group however in an overall low amount (below 5% of days in hospital) and no differences in antidepressants and antipsychotics occurred. Thus, we do not assume that this has affected results.

Furthermore, the present study had a retrospective focus whereas a prospective design should also be pursued in future studies to ensure the treatment success of abbreviated inpatient DBT programs. This could also be used to conduct cost-sensitivity analysis.

## Conclusion

In our retrospective, naturalistic study we showed that an 8 weeks DBT inpatient treatment yielded a significant reduction in BPD symptoms as well as depressive symptoms. No significant differences to a 12 weeks program with equivalent content were found. Accordingly, abbreviated treatments could have a positive effect on costs and benefits compared to the common implementation of 12 week therapy programs. In particular, treatment programs with shorter duration give the opportunity to treat more patients overall in a consecutive time period. This might contribute to better patient-centered care for patients with BPD.

### Supplementary Information

Supplementary Information.

## Data Availability

Aggregated data (e.g. for meta-analysis) will be available from the corresponding author.
