# Supplementary material for: Comparison of 8-vs-12 weeks, adapted dialectical behavioral therapy (DBT) for borderline personality disorder in routine psychiatric inpatient treatment—A naturalistic study
Source: Sci Rep. 2024 May 17;14:11264. doi: 10.1038/s41598-024-61795-9 (PMC11101618; doi:10.1038/s41598-024-61795-9)
Supplement: Supplementary file 1 — Supplementary Information. [file 41598_2024_61795_MOESM1_ESM.pdf]

Supplement

Comparison of 8-weeks pre March 2021, 8-weeks post March 2021 and 12-weeks

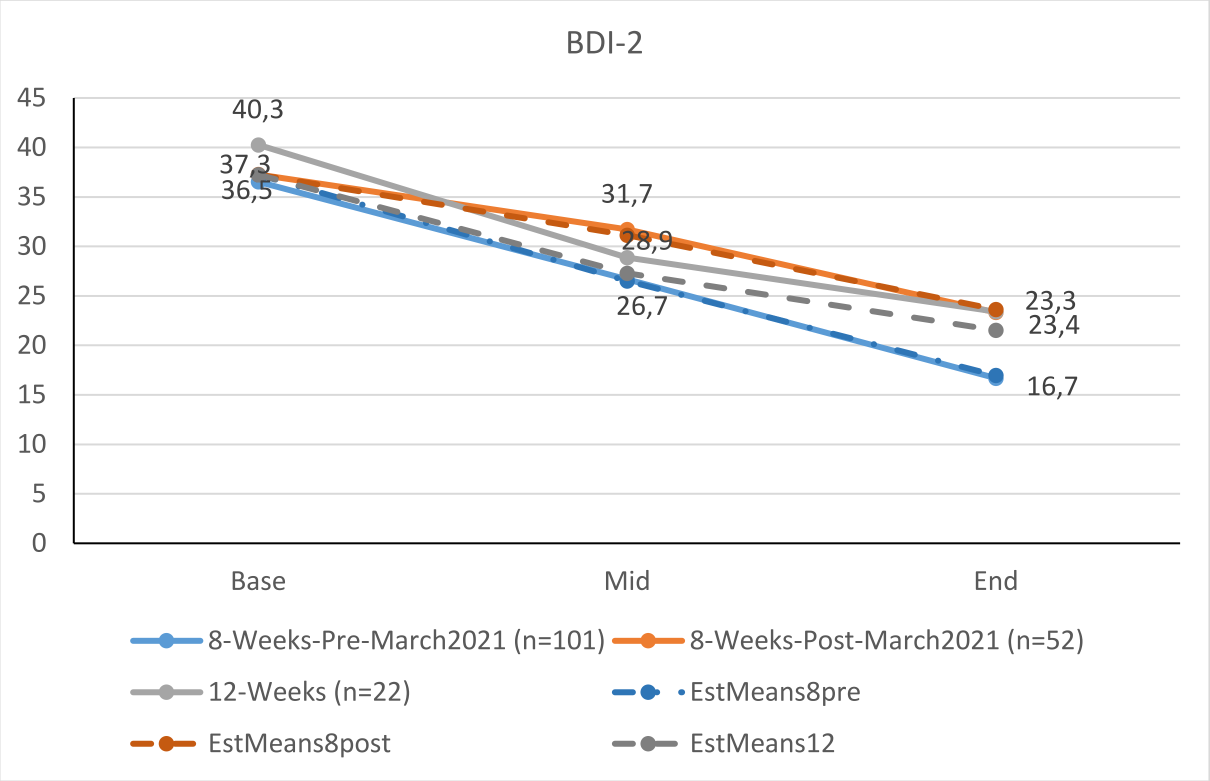

Figure S1. Observed and estimated sum scores for BDI-II for all three groups over time.

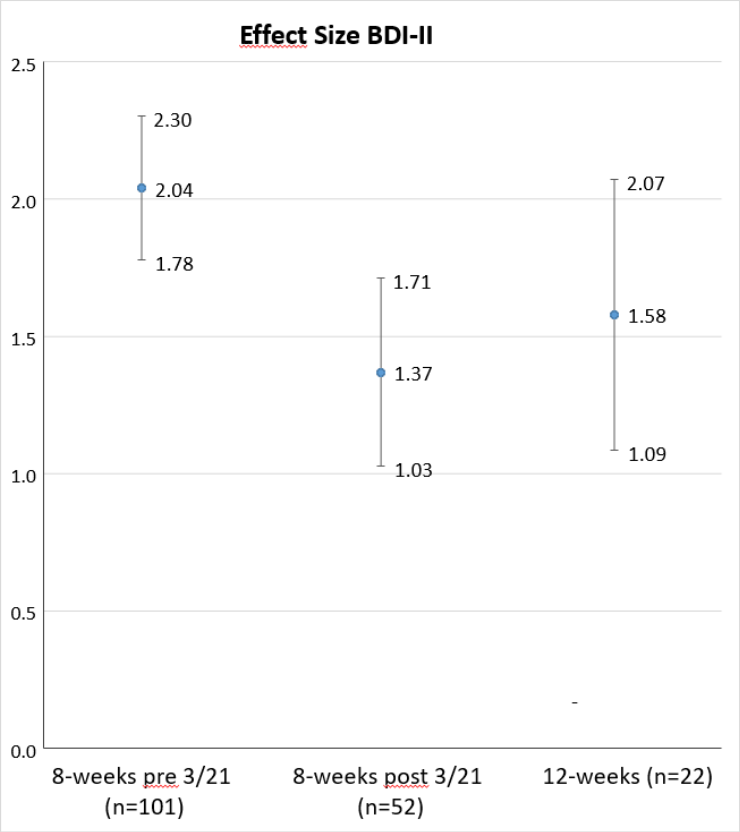

Figure S2. Effect sizes (and 95%-CIs) for 3-group analyses in BDI-II.

Table S1. C*omorbidity with (other) mental disorders according to ICD-10 diagnosis (chapter V: mental and behavioural disorders)*

	**8-weeks**	**12-weeks**	***p***
**Total [N (%)]**	153 (100)	22 (100)	
Any other mental disorder	73 (47.7)	11 (50.0)	.84
Drug abuse disorders (F1x)	24 (15.7)	4 (18.2)	.77
Depression (F32, F33)	132 (86.3)	19 (86.4)	.99
Neurotic, stress-related and somatoform disorders (F4x)	36 (23.5)	4 (18.2)	.58
Other personality disorder than F60.3	14 (9.2)	2 (9.1)	.66
Any other mental disorder not mentioned before	18 (11.8)	2 (9.1)	.99

Table S2. *Treatment with psychotropic drugs within the treatment phase.*

	**8-weeks**	**12-weeks**	***p***
**Rate of patients with specific drugs [N (%)]**			
Any antidepressant (AD)	89 (58.2)	14 (63.6)	.65
SSRIs	49 (32.0)	8 (36.4)	.81
SNRI	17 (11.1)	3 (13.6)	.73
Tricyclic AD	39 (25.5)	4 (18.2)	.60
Other AD	25 (16.3)	7 (31.8)	.14
Any antipsychotic (AP)	89 (58.2)	17 (77.3)	.11
2nd generation APs	33 (21.6)	6 (27.3)	.59
Low-potency (1st generation) APs	70 (45.8)	13 (59.1)	.26
Mood stabilizer	15 (9.8)	2 (9.1)	.92
Benzodiazepine	40 (26.1)	2 (9.1)	.11
Sleeping drugs	18 (11.8)	1 (4.5)	.47
Other psychotropic drugs	8 (5.2)	2 (9.1)	.62
**Days with drugs within treatment phase [mean (SD)]**	**8-weeks**	**12-weeks**	***p***
Any antidepressant (AD)	15.3 (17.7)	24.1 (22.8)	.10
SSRIs	7.3 (13.3)	10.3 (17.3)	.45
SNRI	2.0 (6.9)	7.1 (19.5)	.24
Tricyclic AD	6.6 (14.2)	5.7 (16.6)	.78
Other AD	2.1 (6.9)	7.8 (14)	.08
Any antipsychotic (AP)	13.7 (19.2)	19.4 (21.6)	.20
2nd generation APs	4.6 (11.5)	8.8 (18)	.30
Low-potency (1st generation) APs	9.9 (17.9)	12 (18.6)	.62
Mood stabilizer	2.3 (8.6)	0.1 (0.3)	**<.001**
Benzodiazepine	1.8 (5.9)	1.9 (7.5)	.93
Sleeping drugs	1.7 (6.3)	0 (0.2)	**<.001**
Other psychotropic drugs	0.4 (2.7)	0.1 (0.3)	.55
**% days with drugs within treatment phase [mean (SD)]**	**8-weeks**	**12-weeks**	***p***
Any antidepressant (AD)	27.3 (30.5)	32.0 (29.5)	.50
SSRIs	13.7 (24.7)	13.9 (23.1)	.96
SNRI	3.8 (12.7)	8.9 (23.9)	.34
Tricyclic AD	11.1 (22.8)	7.3 (20.2)	.46
Other AD	3.8 (12.2)	11.3 (20)	.10
Any antipsychotic (AP)	24.1 (32.4)	24.6 (26.8)	.94
2nd generation AP	8.8 (22.4)	10.6 (21.6)	.73
Low-potency (1st generation) AP	17.0 (29.5)	15.7 (24)	.84
Mood stabilizer	4.1 (15.2)	0.1 (0.4)	**<.001**
Benzodiazepine	3.3 (10.9)	2.3 (9.0)	.68
Sleeping drugs	3.3 (12.1)	0.1 (0.3)	**<.001**
Other psychotropic drugs	0.8 (5.3)	0.1 (0.4)	.53
